# An Analysis on the Correlation and Gender Difference between College Students' Internet Addiction and Mobile Phone Addiction in Taiwan

**DOI:** 10.1155/2013/360607

**Published:** 2013-09-17

**Authors:** Shao-I Chiu, Fu-Yuan Hong, Su-Lin Chiu

**Affiliations:** The Central for General Education of Taipei College of Maritime Technology, Taipei 11174, Taiwan

## Abstract

This study is aimed at constructing a correlative model between Internet addiction and mobile phone addiction; the aim is to analyse the correlation (if any) between the two traits and to discuss the influence confirming that the gender has difference on this fascinating topic; taking gender into account opens a new world of scientific study to us. The study collected 448 college students on an island as study subjects, with 61.2% males and 38.8% females. Moreover, this study issued Mobile Phone Addiction Scale and Internet Addiction Scale to conduct surveys on the participants and adopts the structural equation model (SEM) to process the collected data. According to the study result, (1) mobile phone addiction and Internet addiction are positively related; (2) female college students score higher than male ones in the aspect of mobile addiction. Lastly, this study proposes relevant suggestions to serve as a reference for schools, college students, and future studies based on the study results.

## 1. Introduction

The current modes of information and communication technology such as computers, the Internet, and mobile phones have changed adolescents' daily life drastically. In addition to being a convenience to people's communication methods, technology unfortunately has negative side-effects. The most frequent negative side-effect is chronic addiction to technological mediums or excessive human-machine interactions involved. People rely on technological devices to a level of full-blown addiction to obtain pleasure as a psychological benefit. They depend on technology significantly in the hope that it would lessen negative moods or increase positive outcomes [1–4]. According to Griffiths [5], technological addiction is a subcategory of behavioural addiction. He defines it as a behavioural addiction which involves human-machine interaction and is nonchemical in nature. 

However, if speaking from the perspective of substance-based addictions, technological addiction does not produce recognizable signs or features (e.g., the biological indicators of nicotine addiction), and the addicts may develop unacceptable social behaviour and attitude in their daily routines or social life [5, 6]. In this case, there is no denying that technological addiction has caused a negative impact on an individual's life in a harmful manner.

For the time being, studies on Internet addiction and mobile phone addiction are common occurrences. The Internet services and games provided by mobile phones may be considered a way to alleviate loneliness [7]. Besides, a great deal of information can be acquired online to feed or to fuel other addictions or conflicting behaviour. For example, the Internet may have become a highly dangerous medium of porn addiction. 

One must also take into account that some activities like online role playing games may influence Internet users to a higher level of addiction, such as sending and receiving emails, browsing through sites and messages, and uploading or downloading files [8]. Both the Internet and mobile phone addicts are believed to have an unhealthy lifestyle and similar personalities. Moreover, Internet addiction and mobile phone addiction may be closely related [9]. However, studies on analysing the correlation between Internet addiction and mobile phone addiction are rare. Therefore, this study is aimed at discovering the further relationships between these two, to serve as a reference for guiding students' college life.

Gender difference regarding users addicted to the Internet and mobile phones is not only a highly interesting issue but a potential element which can affect the increase of Internet and mobile phone addiction. Although a number of studies have been conducted to discuss this issue, most of them have adopted the Chi-square test to process the data [10, 11], without comparing the differences between individuals' development of Internet and mobile phone addiction simultaneously. Therefore, this study will further analyze the effects of gender differences regarding college students' Internet and mobile phone addiction. Traditional group difference assessment methods like the Chi-square test, *t*-test, or MANOVA may produce false results and cause misunderstanding because they are interpreted based on test scores or composite variables rather than latent variables or factors. 

On the other hand, latent means analysis (LMA) assesses the difference of groups in the manner of a structural equation model, which is effective in terms of controlling measurement errors and the group variance of measurement models. In addition, it may be applied to compare the means of latent structures [12–14]. In this case, this study will establish a model based on the correlation of Internet addiction and mobile phone addiction to discover how male and female college students differ regarding the two sides of the tendency to this technology addiction.

## 2. Literary Review

### 2.1. Internet Addiction and Mobile Phone Addiction

Yen et al. [11] point out addicts to the Internet and to the mobile phone may have similar personalities and lifestyles; there is a significant correlation between the two. First of all, Internet addicts and substance users also tend to have similar personalities. As such, the two may also have related mental illnesses or mechanisms [11]. 

Studies clearly indicate that misuse of the Internet is strongly associated to a number of psychological and behavioural problems. For instance, there exist a great deal of relevance between the misuse of the Internet and issues such as anxiety, depression, loneliness, social isolation, low self-esteem, shyness, abnormal mood swings, precipitated behaviour, and lack of social skills and support [9, 15–24]. 

Likewise, mobile phone addicts tend to be hyper sensitive to interpersonal relationships, and they may have great difficulty communicating with others face to face [25]. In addition, mobile phone addicts are more likely to have characteristics such as hypochondria, maladjusted on social level, sleeping disorders, negative, and/or a low self-esteem, anxiety, depression and introversion [25–28]. Based on the aforementioned reasons, people with Internet addiction and mobile phone addiction may share similar personalities.

Internet addicts may also share parallel lifestyles with mobile phone addicts, making the two positively correlated. Since adolescents addicted to the Internet are more likely to be vulnerable to substance abuse, like alcohol [11, 29], their comorbidity may help to explain why there is a relation between cause and effect and why they have some important factors in common [30]. Moreover, the comorbidity between Internet addiction and alcohol use disorders (AUDs) may imply that the two have relevant mental illnesses or mechanisms [11]. On the other hand, mobile phones are often regarded as a possible competitor regarding nicotine addiction since both fulfil the same need and result in financial exhaustion [31]. However, adolescents do not generally have a lot of money at their disposal. In this case, mobile phones and cigarettes are both regarded as substitutes [32] or a supplement. It will depend on the sharing of identical lifestyle features [33, 34]. 

Studies suggest that there is a positive correlation between the excessive use of mobile phones and unhealthy behaviours like smoking and drinking [10, 34, 35]. Furthermore, smokers may become heavy mobile phone users [34]. This study hypothesizes that there is a positive correlation between Internet usage, mobile phone addiction, and unhealthy behaviours. More specifically, Internet addiction and mobile phone addiction may share similar impact factors and mechanisms; thus, the two can be correlated with each other [9].

### 2.2. Gender and Internet and Mobile Phone Addiction

With regards to the literary reviews of previous studies on the gender differences between Internet addiction and mobile phone addiction, there is no consistent conclusion yet. According to the studies adopting the Chi-square test for analysis, male college students are more prone to Internet addiction than female ones [11, 29]. Likewise, Gnisci et al. [36] adopted the point-biserial correlation analysis and concluded that male college students are more likely to be dependent on the Internet than their female counterparts. Some studies suggest that Internet addicts are primarily shy teenage boys, but the number of teenage girls addicted to the Internet is increasing [37, 38]. However, according to the studies of Chang and Law [39] and Beranuy et al. [9], the result of multivariate analysis of variance (MANOVA) implies that Internet addiction does not show a significant difference between genders.

Studies also indicate that females may be more likely to develop mobile phone dependency [40], mobile phone abuse [9], mobile phone involvement [41], and mobile phone addiction [42]. More specifically, Jenaro et al. [28] argues that 28.6% of all male college students and 56.3% of all female college students are classified as heavy mobile phone users. To sum up, the literature seems to suggest that male college students tend towards Internet addiction, while their female counterparts seem to develop an addiction to mobile phones. There is still a great deal of disagreement among various studies. This data also fails to make a conclusive gender difference comparison between Internet addiction and mobile phone addiction. This study will further analyze this issue.

## 3. Materials and Methods

### 3.1. Participants

The participants of this study were primarily selected from two colleges on the island for convenient sampling, Taipei College of Maritime Technology and Aletheia University. The study issued 500 copies of questionnaires for the purpose of the survey and collected data for two weeks. The test time of the questionnaire is approximately 10 minutes. After collecting all the questionnaire copies and removing the blank and invalid ones, the study acquired 448 valid copies, with 238 of them coming from the Taipei College of Maritime Technology (53.1% of the study) and 210 of them from the Aletheia University (46.9% of the study). Concerning the year of study of the participants, 196 of them were freshmen (43.8%), 120 were sophomores (26.8%), 92 were junior students (20.5%), and 40 were senior students (8.9%). Regarding the gender of participants, 274 of them were males (61.2%) and 174 were females (38.8%).

### 3.2. Measures

This study adopted the Mobile Phone Addiction Scale (MPAS) developed by Hong, Chiu, and Huang (in press) to serve as the measurement tool. The scale score is designed in accordance with the 6-point Likert Scale (from 1 = “completely disagree” to 6 = “complete agree”). This scale is suitable for evaluating the three psychological features of mobile phone addiction: firstly, time management (5 questions), in which participants may be asked if they will think about using “a couple of more minutes” when they are on the mobile phone. Secondly, school performance (3 questions), in which participants may be asked if their school performance will be affected because they spend too much time on mobile phone. Thirdly, substitute satisfaction (3 questions), in which participants may be asked if they will check their mobile phone to make sure they do not miss any calls or messages before they have to do other things. The internal consistency reliability among the three scale aspects is 0.84, 0.89, and 0.77, respectively, making the overall internal consistency Cronbach's *α* = 0.91, which indicates that the scale is reliable. 

Regarding the measurement of Internet addiction, the study adopts the Chinese Internet Addiction Scale designed by Cheng et al. [43] to explore the phenomenon of college students' Internet addiction. The scale score is developed based on 6-point Likert Scale (from 1 = “completely disagree” to 6 = “completely agree”). The scale is also divided into four aspects: tolerance of Internet addiction (6 questions), compulsive Internet use and withdrawal syndrome from Internet addiction (6 questions), interpersonal relationship and health issues (5 questions), and time management (7 questions). Participants will be asked 24 questions in total, including “I have tried to spend less time on the Internet, but I cannot make it,” “I feel uneasy as long as I am away from the Internet for a while,” “Because of the Internet, my interaction with family members and friends has become less,” “There was more than once that I slept for less than 4 hours due to the Internet.” The internal consistency reliability among all scale aspects is 0.90, 0.90, 0.88, and 0.84, making the overall internal consistency Cronbach's *α* = 0.95, which indicates that this scale is greatly reliable.

### 3.3. Data Analysis

This study is aimed at discussing the relation between Internet addiction and mobile phone addiction, and how college students of different genders vary in the means of the two so as to construct a correlation model of Internet and mobile phone addiction. The variables of Internet addiction in the model include the tolerance of Internet addiction, compulsive Internet use and withdrawal syndrome from Internet addiction, interpersonal relationship and health issues, and time management. Those of mobile phone addiction include time management, school performance, and substitute satisfaction. 

First, the study adopts descriptive statistics to analyze college students' self-reported situations of Internet addiction and mobile phone addiction. Then, the study selects a *t*-test to analyse how male and female students vary in various scale aspects and the overall scores, then they adopt bivariate correlations to assess the relation between Internet addiction and mobile phone addiction. Lastly, in order to understand how male and female college students differ in the latent means of Internet addiction and mobile phone addiction, the study analyses the invariance of the model before comparing the appropriateness of the fit of the latent mean structural modelling. 

The study adopts the maximum likelihood approach to conduct the parameter estimation. As for the appropriate fit index, it includes *χ* ^2^, GFI, CFI, and RMSEA. The standard of *χ* ^2^/DF is less than 3 [44], while the index of GFI and TLI has to exceed 0.9 [45]. The index of CFI has to exceed 0.95 [46] and that of RMSEA has to be less than 0.08 [47]. In this case, the appropriate fit is considered acceptable.

## 4. Results

### 4.1. Descriptive Statistics

According to the normal distribution examination, it was discovered that the skewness and the kurtosis of the following nine variables were all less than 2, indicating that the collected data was normally distributed: they were “time management,” “school performance,” “substitute satisfaction,” and the overall score in the section of mobile phone addiction and “tolerance of Internet addiction,” “compulsive Internet use and withdrawal syndrome from Internet addiction,” “interpersonal relationship and health issues,” “time management,” and the overall score in the section of Internet addiction. 

The analysis of male and female college students' mobile phone addiction and Internet addiction in terms of various scale aspects and the overall score is shown in Table 1. When speaking from the perspective of the nine aforementioned aspects and the overall scores, college students' mobile phone and Internet addiction do not show any difference due to gender. Nevertheless, female college students tend to have more time management issues related to mobile phone addiction than male ones, and they score higher than male students in terms of the overall score of mobile phone addiction.

### 4.2. Correlations between Variables

The means, standard deviation, and correlation coefficient of the variables are shown in Table 2. According to the analysis concerning the data of male college students, there is a significant positive correlation among all variables, and the correlation coefficients of “time management,” “school performance,” and “substitute satisfaction” in the aspect of mobile phone addiction along with “compulsive Internet use and withdrawal syndrome from Internet addiction,” “interpersonal relationship and health issues,” and “time management” in the aspect of Internet addiction are all above 0.26. The correlation coefficients of “time management” and “school performance” of mobile phone addiction and the “tolerance of Internet addiction” of Internet addiction are 0.14 and 0.13, respectively, which are relatively low. 

Based on the data analysis of female college students, the correlation coefficients of all variables suggest that each variable is significantly positively correlated. The correlation coefficients of “time management” and “substitute satisfaction” in the aspect of mobile phone addiction along with “tolerance of Internet addiction,” “compulsive Internet use and withdrawal syndrome from Internet addiction,” “interpersonal relationship and health issues,” and “time management” in the aspect of Internet addiction are all above 0.22. Those of “school performance” in the aspect of mobile phone addiction along with “compulsive Internet use and withdrawal syndrome from Internet addiction,” “interpersonal relationship and health issues,” and “time management” in the aspect of Internet addiction are all above 0.21. There is no correlation between “school performance” of mobile phone addiction and the “tolerance of Internet addiction” of Internet addiction. All things considered, the hypothetical model of this study is worth further analysing.

### 4.3. Assessing the Model Fit

In the hypothetical model developed by this study, there is a positive significant correlation between mobile phone addiction and Internet addiction. Since the acquired appropriateness of this fit is *χ* ^2^ (13, *n* = 448) = 10.175, *P* = 0.000, GFI = 0.916,CFI = 0.930, RMSEA = 0.143, it indicates that the fit is not acceptable and that modification is needed. After reviewing the modification indices (MI), it was discovered that the MI between “tolerance of Internet addiction” and “compulsive Internet use and withdrawal syndrome from Internet addiction” reaches 60.71 and that the adjustable parameter is 8.71. 

**Figure 1:**
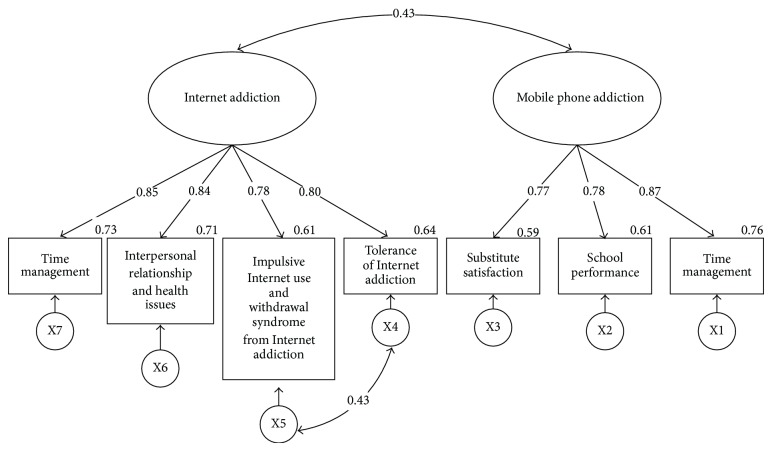
Model on the correlation between male college students' Internet addiction and mobile phone addiction. Note: inside the rectangle are the variables of measurement; all values are standardized parameter estimations.

**Figure 2:**
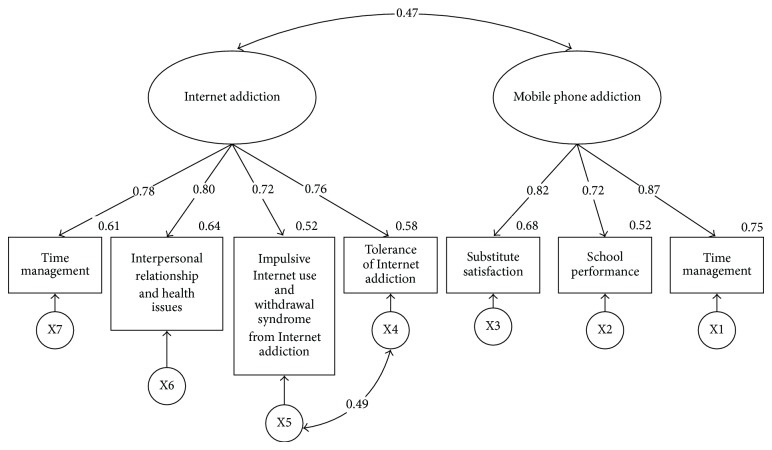
Model of the correlation between female college students' Internet addiction and mobile phone addiction. Note: inside the rectangle are the variables of measurement; all values are standardized parameter estimations.

After freeing the covariance parameter of the two variables, it was discovered that the MI of model II is *χ* ^2^ (12, *n* = 448) = 3.301, *P* < 0.000, GFI = 0.976, CFI = 0.984, RMSEA = 0.072. Except for the Chi-square test, which does not fit, the rest of the model fits, which indicates that this structural model of mobile phone addiction and Internet addiction can be applied to test the samples of college students. Afterwards, the analysis of invariance modelling and means structure shall be conducted based on this modified model II, show in Table 3(a) and the models of male and female college students are shown in Figures 1 and 2, respectively.

In order to understand the gender differences between mobile phone addiction and Internet addiction, the study firstly limits the error variance of male and female college students' observable variables and unobservable latent to be 0 as the baseline model (model III) show in Table 3(a) and conducted the invariance analysis of factor loading, covariance, and residual [12, 13, 48]. 

The study limits the factor loading of male and female students to be equal and names it the invariance model of the factor loading (model IV), while the factor loading and covariance are limited to be equal as the invariance model of structural covariance (model V). The third model, the residual invariance model (model VI), has an equal factor loading, covariance, and residual show in Table 3(a). 

**Figure 3:**
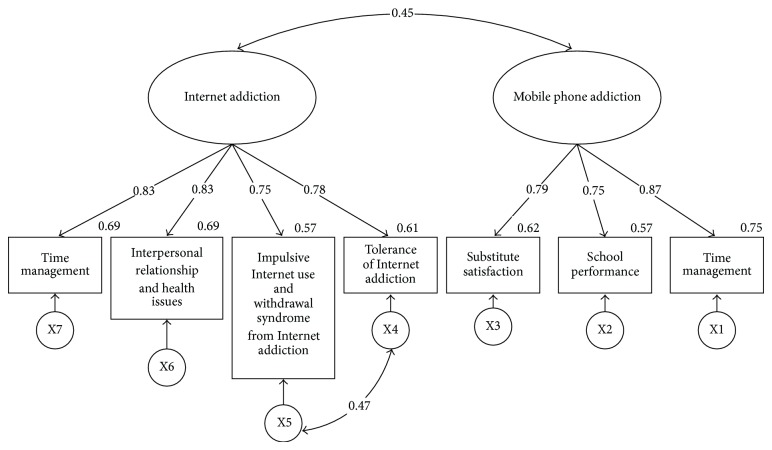
Invariance model of variable intercepts of college students' Internet addiction and mobile phone addiction. Note: inside the rectangle are the variables of measurement; all values are standardized parameter estimations.

Afterwards, the study conducts a model comparison to discover if the factor loading, covariance, and residual of the model of male and female college students' mobile phone addiction and Internet addiction has an invariance feature. The comparative results are shown in Figure 3. Lastly, the study compared the latent mean structural models of males and females, respectively, to determine their appropriate fit. 

According to the invariance analysis show in Table 3(a), model III and IV, model IV and V, and model V and VI show no differences in the Chi-square test with CFI and TLI being above 0.95, which are acceptable. The indices of RMSEA are below 0.08, and the smallest value of ECVI appears in the residual invariance model (model VI), indicating that the factor loading, covariance, and residual of the relation model of mobile phone addiction and Internet addiction are in invariance. 

In the residual invariance model of male college students, there is a positive significant correlation (*r* = 0.43, *P* < 0.001) between mobile phone addiction and Internet addiction. Likewise, in the residual invariance model of female college students, there is also a positive significant correlation (*r* = 0.47, *P* < 0.001) between mobile phone addiction and Internet addiction.

The aforementioned invariance models of factor loading, covariance, and residual are a fit. Afterwards, the study is going to examine the latent means of different genders. It is necessary to ensure the invariance of observed variables' intercepts beforehand. This study constrains the factor loading, covariance, variance, the error of means, and the intercepts of variables to all be 0 as the baseline model (the invariance model of variable intercept, model VII). 

The obtained fit indices are *χ*_(50)_^2^  (*N*_male_ = 274,  *N*_female_ = 174) = 3.421, *P* < 0.001, CFI = 0.929, TLI = 0.941, RMSEA = 0.047, indicating that the model is a fit. However, when comparing the invariance model of variable intercepts and the residual model, it was discovered that *χ*_(10)_^2^ = 1.543 and *P* > 0.05, indicating that the intercepts were invariant. In this case, the study makes model VIII a baseline model. The latent mean analysis of model VIII is shown in Figure 3. First of all, the study hypothesizes that the latent means of male and female college students' mobile phone addiction are equal to model IX, and the appropriateness of the fit index will be *χ*_(51)_^2^  (*N*_male_ = 274,  *N*_female_ = 174) = 3.494, *P* < 0.001, CFI = 0.926, TLI = 0.93 and RMSEA = 0.075, which indicates that this model fits. 

If compared with the baseline model, *χ*_(1)_^2^ = 7.181 and *P* < 0.01, indicating that male and female college students' scores of mobile phone addiction are not equal. Therefore, the study examines the average scores of male and female college students' mobile phone addiction as 9.842 and 11.769, respectively, suggesting that female college students are more addicted to mobile phone than male ones. 

The study also hypothesizes that the latent means of male and female college students' Internet addiction are equal; as model X and appropriateness of fit will be *χ*_(51)_^2^  (*N*_male_ = 274,  *N*_female_ = 174) = 3.369, *P* < 0.001, CFI = 0.929, TLI = 0.942 and RMSEA = 0.073, which indicates that this model fits. If compared with the baseline model, *χ*_(1)_^2^ = 0.767 and *P* > 0.05, indicating that male and female college students' scores of Internet addiction are equal. 

## 5. Discussion

For the time being, Internet addiction and mobile phone addiction are two of the most commonly discussed technological addictions. Beranuy et al. [9] suggest that there is a positive significant correlation existing between the score of the Mobile Phone Addiction Scale and that of the Internet Addiction Scale. The reason for this may be that the two have similar personalities and lifestyles. This study adopted the different scale structures to analyze the correlation between Internet addiction, and mobile phone addiction, and the study result is beneficial to improving people's understanding of the relations between college students' mobile phone addiction and Internet addiction. It also supports the study result of Beranuy. 

Moreover, this study adopted the latent means analysis of the structural equation model to compare the means difference between male and female college students in terms of Internet addiction and mobile phone addiction, in order to support the hypothesis that there may be a gender difference in Internet and mobile phone addiction. 

Firstly, according to the results of the study, it was discovered that mobile phone addiction and Internet addiction are significantly positively correlated, which conforms to the study of Beranuy et al. [9]. In other words, the technological addiction of college students has gone beyond the Internet; they are addicted to mobile phones as well. Because of their technological addiction, college students tend to indulge themselves on the Internet and on mobile phones, and they may experience depression and negative emotions if they do not have them. 

That is to say, technological addiction has a great impact on college students in terms of their mental health, time allocation and management, school performance, interpersonal relationships, and health. It was also shown that there is no significant difference in gender between college students' mobile phone addiction and Internet addiction. For both male and female college students, the more addicted to the Internet they are, the higher the possibility of becoming addicted to mobile phones. 

The main reason may be that mobile phones share similar functions with the Internet. For example, both of them allow users to send messages as a means to interact with others via Internet services and provide users (viz, college students) with games to kill time. The correlation between Internet addiction and mobile phone addiction also suggests that the two may feed and fuel each other. When college students cannot use computers, they are very likely to turn to mobile phones to satisfy their demands of Internet services and games. That is to say, the Internet and mobile phone can be substituted in terms of satisfaction. 

It was also discovered that there was a significant relation between the time which college students spend on the Internet and that spent on mobile phones. In other words, the longer a college student spend time on the Internet, the more likely he/she will use the mobile phone with Internet services to satisfy his/her addiction caused by excessive Internet use. Perhaps those with better tolerance of Internet addiction may select mobile phones to satisfy their yearning and thus develop the condition of substitute satisfaction. If this viewpoint is acceptable, it depends on future studies to discover substitute satisfaction between Internet addiction and mobile phone addiction in the manner of longitudinal studies.

This study adopted the latent means of multisample structural equation modelling, which is effective in controlling measurement errors and the group variance of measurement models as the analysis tool and concluded that Internet addiction does not show any difference because of gender. This study result is very similar to that of Beranuy et al. [9] and of Chang and Law [39] though most studies suggest that male college students have a greater possibility of becoming Internet addicts [11, 29, 36]. 

Regarding online information, females favour online communication and personal messages, while males concern themselves with weather, sports, and games [49, 50]. The boom of social network services attracts more females to use the Internet, and their daily lives may be seriously affected [51]. In this case, male and female college students show little difference in terms of Internet addiction. 

It was discovered that female college students tend to score higher than their male counterparts in the aspect of mobile phone addiction, which is in agreement with previous studies [9, 28, 40–42]. The reason may be that female college students are more likely to maintain their social relationships and communicate with the people they value via mobile phone. Moreover, based on the study result, a high percentage of female college students like to make phone calls at night and text people daily. In other words, they prefer the manner of indirect communication [10, 26, 40, 52–54]. In addition, female college students favour staying in touch with people and communicating with family members via email to establish close relationships [49, 55].

Though this study has obtained two important results, namely, that the correlation between Internet addiction and mobile phone addiction is positive and female college students are more addicted to mobile phone than male ones, there are still some restrictions needed to be taken into consideration when interpreting the findings of the study. 

First of all, a high percentage of the study's participants are freshmen. Also, there were more male participants than female ones, making this study somewhat weak in terms of representation. Secondly, both the Internet addiction and mobile phone addiction surveyed by this study do not reach the extent of clinical level, and the correlation between the two types of addiction can only reflect the preliminary study on how college students use the Internet and mobile phone. As for the substitution of mobile phones for the Internet, it will still need further studies to conduct analysis in the manner of experiment and longitudinal studies.

This study result is also a good reference for future studies. Since this study is aimed at discussing the correlation and gender differences between college students' Internet addiction and mobile phone addiction and concludes that the two may share similar personalities and lifestyles, it is worth exploring whether these personalities and lifestyles will strengthen or weaken the two types of technological addictions. On the other hand, it is also worth discussing if a college student has to have a certain personality to become both an Internet and a mobile phone addict, which can serve as a reference to identify technological addiction and guide college students. Lastly, future studies are advised to design a technological addiction scale based on this study result to serve as a tool to identify the negative effects of technological development on adolescents in the future.

## Figures and Tables

**Table 1:** Summary of the means, standard deviation, and range of variables.

	Male	Female	Male	Female	*t*
	Skew/Kurtosis	Skew/Kurtosis	Mean ± SD	Mean ± SD	
Time management (MPA)	1.19/1.29	0.42/−0.76	9.84 ± 5.17	11.71 ± 5.38	−3.68∗∗∗
School performance	1.69/2.47	1.29/1.15	5.11 ± 3.27	5.36 ± 3.16	−0.82
Substitute satisfaction	0.61/−0.37	0.21/−0.57	7.42 ± 3.95	7.99 ± 3.39	−1.64
Total scale	1.16/1.39	0.54/−0.40	22.36 ± 10.87	25.07 ± 10.59	−2.60∗∗

Tolerance of Internet addiction	0.01/−0.66	−0.22/−0.49	20.05 ± 7.83	20.48 ± 7.41	−0.58
Impulsive Internet use and withdrawal syndrome from Internet addiction	0.47/−0.10	0.37/−0.33	16.45 ± 7.12	17.03 ± 7.02	−0.85
Interpersonal relationship and health issues	0.29/−0.62	0.45/−0.11	13.66 ± 6.11	12.89 ± 5.57	1.34
Time management (IA)	0.39/−0.31	0.67/−0.11	18.91 ± 7.95	17.84 ± 6.69	1.49
Total scale	0.16/−0.24	0.16/−0.22	69.06 ± 25.33	68.24 ± 22.54	0.35

^**^ *P* < 0.01; ^***^ *P* < 0.001.

**Table 2:** Summary of the correlation coefficient of mobile addiction and Internet addiction (*n*_male_ = 274, *n*_female_ = 174).

	1	2	3	4	5	6	7
(1) Time management (MPA)	1	0.74∗∗∗	0.61∗∗∗	0.14∗	0.27∗∗∗	0.32∗∗∗	0.35∗∗∗
(2) School performance	0.70∗∗∗	1	0.60∗∗∗	0.13∗	0.26∗∗∗	0.31∗∗∗	0.31∗∗∗
(3) Substitute satisfaction	0.68∗∗∗	0.62∗∗∗	1	0.28∗∗∗	0.30∗∗∗	0.29∗∗∗	0.34∗∗∗
(4) Tolerance of Internet addiction	0.27∗∗∗	0.15	0.25∗∗∗	1	0.76∗∗∗	0.67∗∗∗	0.63∗∗∗
(5) Impulsive Internet use and withdrawal syndrome from Internet addiction	0.38∗∗∗	0.21∗∗	0.27∗∗∗	0.74∗∗∗	1	0.66∗∗∗	0.64∗∗∗
(6) Interpersonal relationship and health issues	0.40∗∗∗	0.32∗∗∗	0.25∗∗∗	0.61∗∗∗	0.61∗∗∗	1	0.76∗∗∗
(7) Time management (IA)	0.34∗∗∗	0.28∗∗∗	0.22∗∗	0.48∗∗∗	0.47∗∗∗	0.70∗∗∗	1

Note: the lower left value is the correlation coefficient of female college students, while the upper right value is that of male college students.

^*^ *P* < 0.05; ^**^ *P* < 0.01; ^***^ *P* < 0.001.

**Table:** (a)

Model	Chi-square test	Degree of freedom (df)	Chi-square test/df	CFI	TLI	RMSEA	ECVI
Males	32.900	12	2.742 (*P* = 0.001)	0.981	0.967	0.080	0.238
Females	18.766	12	1.564 (*P* = 0.094)	0.989	0.981	0.057	0.293
Baseline model (model III)	51.663	24	2.153 (*P* = 0.001)	0.984	0.972	0.051	0.322
Invariance model of factor loading (model IV)	58.506	29	2.017 (*P* = 0.001)	0.983	0.972	0.048	0.315
Invariance model of structural covariance (model V)	62.063	32	1.939 (*P* = 0.000)	0.982	0.977	0.046	0.310
Residual invariance model (model VI)	75.128	40	1.878 (*P* = 0.001)	0.979	0.978	0.044	0.303

**Table:** (b)

Model comparison	Difference of Chi-square	df	*P* value
Model III and VI	6.842	5	0.233
Model IV and V	3.272	3	0.352
Model V and VI	13.668	8	0.091
